# Supplementary material for: Roles of the zona pellucida in gamete fusion and of the perivitelline space in blocking polyspermy in mice
Source: EMBO Rep. 2025 Dec 8;27(3):774–92. doi: 10.1038/s44319-025-00670-8 (PMC12894720; doi:10.1038/s44319-025-00670-8)
Supplement: Supplementary file 1 — Appendix [file 44319_2025_670_MOESM1_ESM.pdf]

Appendix

Table of contents

Appendix S1- Kinetics of sperm penetration in the PVS of unfertilized oocytes and natural baseline permeability of the ZP……………………………………………………………………………….p2

Appendix Figure S1- Kinetics of sperm penetration in the PVS of unfertilized oocytes………………p2

Appendix S2- Kinetics of sperm engulfment and second polar body release………………………….p3

Appendix Figure S2- Kinetics of sperm engulfment after fusion and release of the second polar body.p3

S1-Kinetics of sperm penetration in the PVS of unfertilized oocytes and natural baseline permeability of the ZP

Among the 93 oocytes that underwent in vitro fertilization with kinetic tracking (Condition 3 in Figure 2A), 14 failed to fertilize, even though at least one, and often several, spermatozoa successfully reached their perivitelline space (PVS) (Figure S1A). Although this failure to fertilize highlights a defect in the fusion process itself, it also demonstrated that spermatozoa can traverse the ZP of these oocytes. Whatever they were the first or the last to penetrate, all spermatozoa passed through a ZP that had not undergone any modification resulting from fertilization. These penetrated but unfertilized oocytes therefore provide a valuable model to investigate whether sperm penetration alone can influence ZP permeability. As for the fertilized oocytes, we identified a penetration time window for the 46 spermatozoa that reached the PVS of these 14 unfertilized oocytes (Figure S1A). To assess the potential impact of sperm penetration on the ZP properties, we plotted the average number of sperm entering the PVS of an oocyte as a function of time after a first penetration (blue dark dots in Figure S1B). This number increases linearly over time (light blue line in Figure S1B), indicating a constant rate of sperm penetration (purple line in Figure S1B). The best linear fit yielded a steady penetration rate equal to $1.28 \pm 0{,}07$ sperm/oocyte/hour). Notably, the similarity between this value and the penetration rate at time of first fertilization of fertilized oocytes (1.21 ± 0.37 sperm/oocyte/hour) confirms that the ZP of the unfertilized oocytes maintain normal permeability, while also providing a quantitative reference for the natural baseline permeability of the ZP.

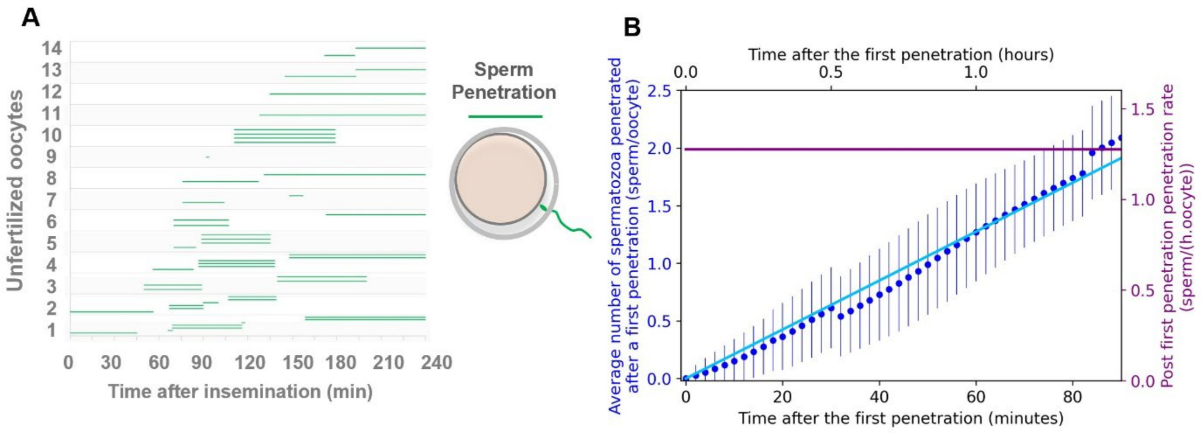

Appendix Figure S1- Kinetics of sperm penetration in the PVS of unfertilized oocytes A- *Chronogram of penetration events in unfertilized oocytes (14 oocytes, 46 penetrated spermatozoa). Each strip corresponds to one oocyte. Each green line within one strip represents the penetration time window of an unfertilizing spermatozoon.* **B**- *Average number of penetrated spermatozoa per unfertilized oocyte (dark blue dots, error bars: mean$\pm 1.96 SEM$) that penetrated after a first penetration obtained by statistical analysis of unfertilized oocytes' penetration chronogram (Figure S1B). It is fitted by a linear function (light blue line) showing a steady penetration rate equal to* 1.28 ± 0.07 *sperm/oocyte/hour (purple line). The steady penetration rate shows that penetration has no influence on ZP permeability to spermatozoa.*

S2- Kinetics of sperm engulfment and second polar body release

Fusion initiates the resumption of meiosis, during which the spermatozoon head is engulfed into the ooplasm and the second polar body (PB2) is extruded, marking the completion of meiosis (Figure S2A). Unlike in vivo and standard in vitro fertilizations (Conditions 1 and 2 in Figure 2A), where these events occur without direct visualization, bright-field in vitro fertilization imaging with kinetic tracking (Condition 3 in Figure 2A) allows for real-time monitoring of their progress. In bright field imaging, sperm engulfment is characterized by the gradual loss of the visibility if the sperm head at the oolemma, while PB2 extrusion is observed as the progressive emergence of a rounded protrusion from the oocyte membrane. Eighteen fertilization events for which the fertilization time could accurately be evaluated, enabled us to establish average kinetics for both sperm engulfment and PB2 release (Figure S2). Sperm engulfment culminates in the complete disappearance of the sperm head approximately 24 ± 3 minutes after the cessation of its movement at the oolemma (Figures S2B and S2C). PB2 release begins shortly thereafter, typically 28 ± 2 minutes after arrest, with the protrusion forming a 90° contact angle with the oolemma by 49± 6 min, and full extrusion completed at approximately 73 ± 10 minutes following the initial cessation of sperm oscillations (Figures S2B and S2D).

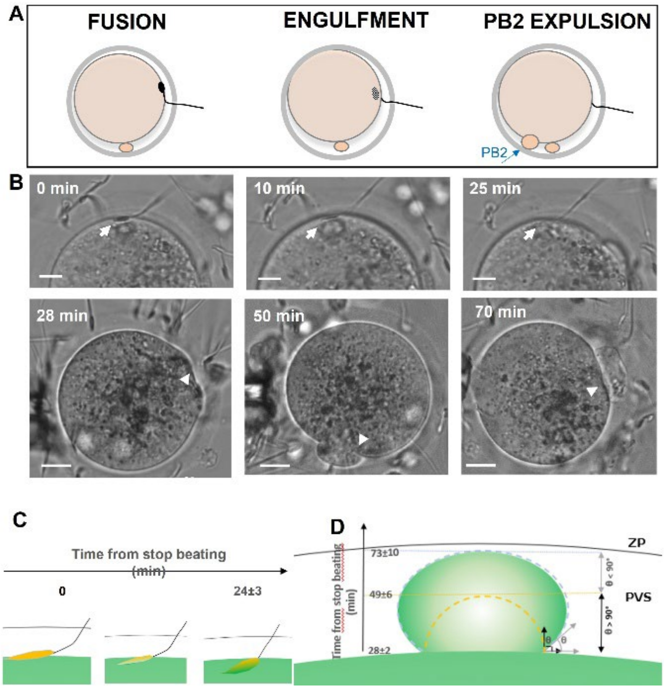

Appendix Figure S2- Kinetics of sperm engulfment after fusion and release of the second polar body A -*Post-fertilization resumption of meiosis: engulfment of the spermatozoon head in the ooplasm, expulsion of the second polar body (PB2) marking the end of the meiosis* **B-** *Bright field pictures illustrating sperm engulfment (top row, white arrows) and subsequent PB2 expulsion (bottom row, white head arrows). While engulfed into the ooplasm, the visibility of the spermatozoon head gradually decreases until, once fully engulfed it becomes indistinguishable. Only the sperm flagellum remains visible, straight and rigid, like a needle stuck in the oolemma. Scale bars: 10 µm.* **C-** *Kinetics of sperm engulfment: on average the fertilizing spermatozoon is fully engulfed into the ooplasm 24±3 min (mean ±SD) after fusion characterized by the arrest of sperm head movement on the oolemma.* **D-** *Kinetics of the PB2 expulsion: on average PB2 expulsion begins 28±2 min (mean ±SD), forms a 90° contact angle with the oolemma by 49± 6 min (mean ±SD) and is complete 73 ±10 min (mean ±SD) after sperm movement arrest.*
