# Supplementary figures and images for: Roles of the zona pellucida in gamete fusion and of the perivitelline space in blocking polyspermy in mice
Source: EMBO Rep. 2025 Dec 8;27(3):774–92. doi: 10.1038/s44319-025-00670-8 (PMC12894720; doi:10.1038/s44319-025-00670-8)

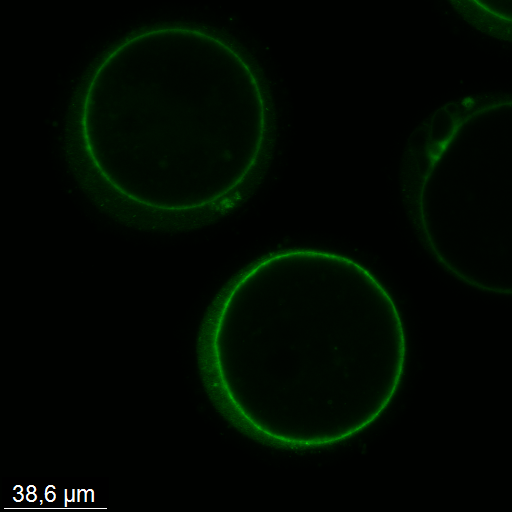

Supplement: Supplementary file 11 — Source data Fig. 6 [file 44319_2025_670_MOESM11_ESM.zip › Fig 6/Figure 6A/ovo 3NF 5F 10Hz_ch00.tif]

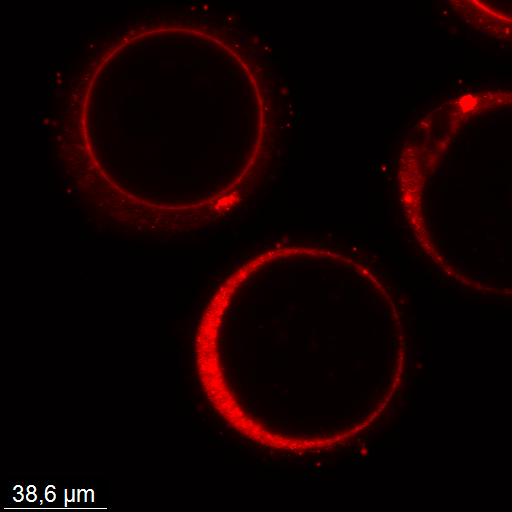

Supplement: Supplementary file 11 — Source data Fig. 6 [file 44319_2025_670_MOESM11_ESM.zip › Fig 6/Figure 6A/ovo 3NF 5F 10Hz_ch01.tif]

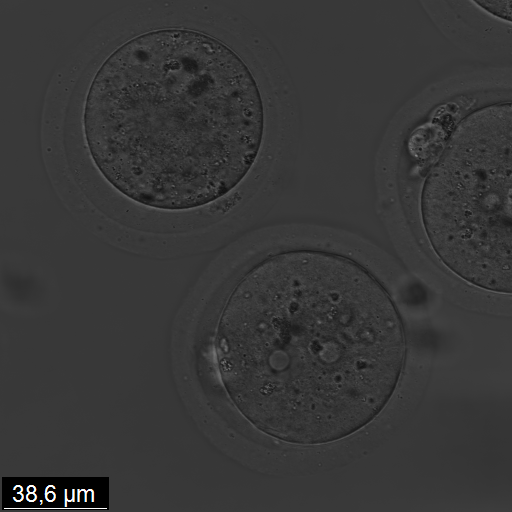

Supplement: Supplementary file 11 — Source data Fig. 6 [file 44319_2025_670_MOESM11_ESM.zip › Fig 6/Figure 6A/ovo 3NF 5F 10Hz_ch02.tif]

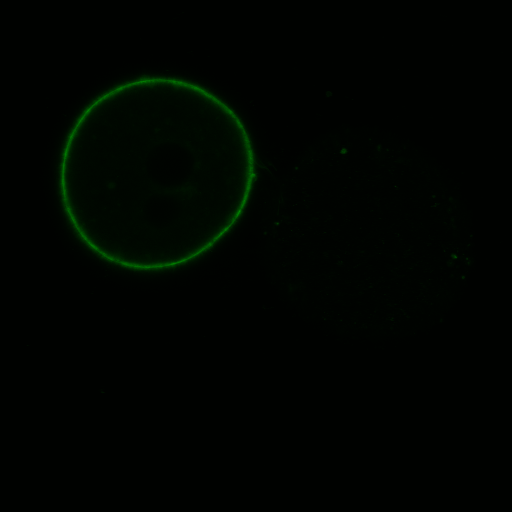

Supplement: Supplementary file 11 — Source data Fig. 6 [file 44319_2025_670_MOESM11_ESM.zip › Fig 6/Figure 6A/ovo 5F depellucide 10 Hz_ch00.tif]

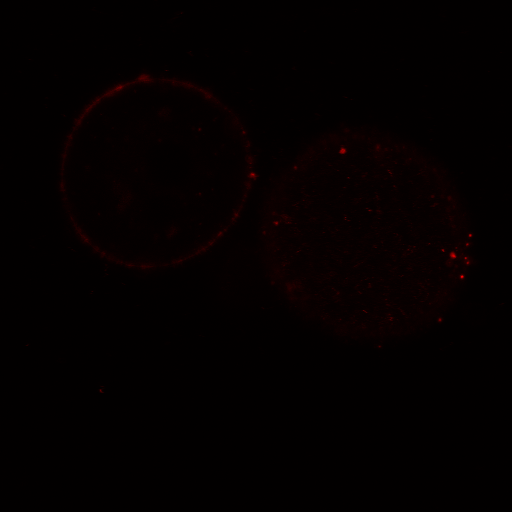

Supplement: Supplementary file 11 — Source data Fig. 6 [file 44319_2025_670_MOESM11_ESM.zip › Fig 6/Figure 6A/ovo 5F depellucide 10 Hz_ch01.tif]

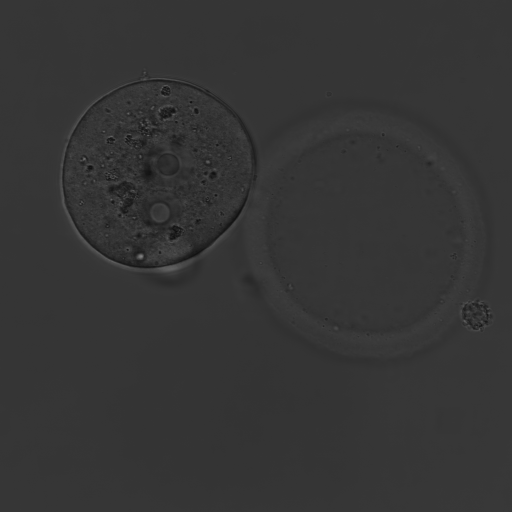

Supplement: Supplementary file 11 — Source data Fig. 6 [file 44319_2025_670_MOESM11_ESM.zip › Fig 6/Figure 6A/ovo 5F depellucide 10 Hz_ch02.tif]

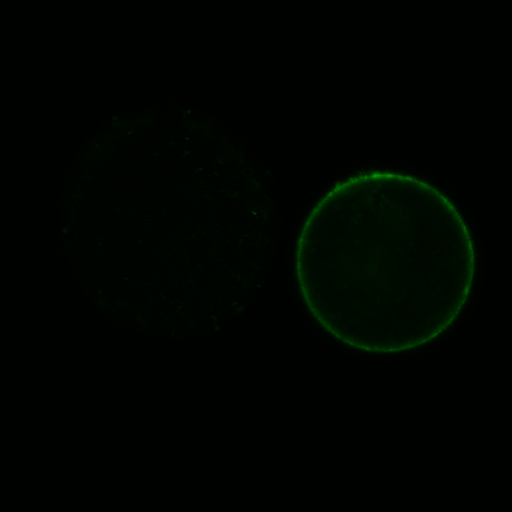

Supplement: Supplementary file 11 — Source data Fig. 6 [file 44319_2025_670_MOESM11_ESM.zip › Fig 6/Figure 6A/ovo3NFdepellucide 10Hz _ch00.tif]

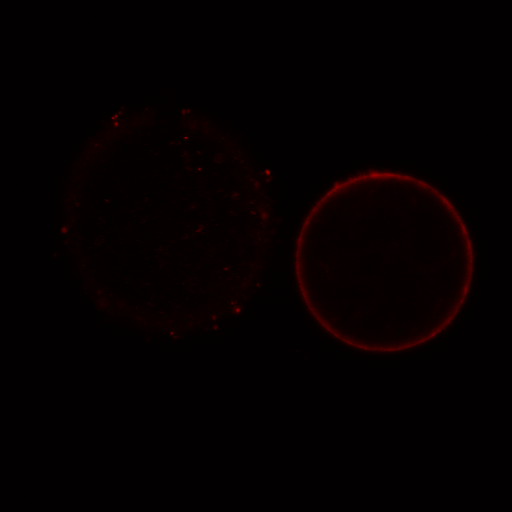

Supplement: Supplementary file 11 — Source data Fig. 6 [file 44319_2025_670_MOESM11_ESM.zip › Fig 6/Figure 6A/ovo3NFdepellucide 10Hz _ch01.tif]

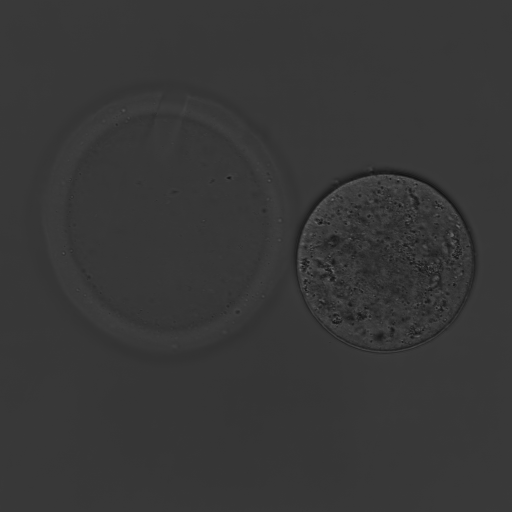

Supplement: Supplementary file 11 — Source data Fig. 6 [file 44319_2025_670_MOESM11_ESM.zip › Fig 6/Figure 6A/ovo3NFdepellucide 10Hz _ch02.tif]

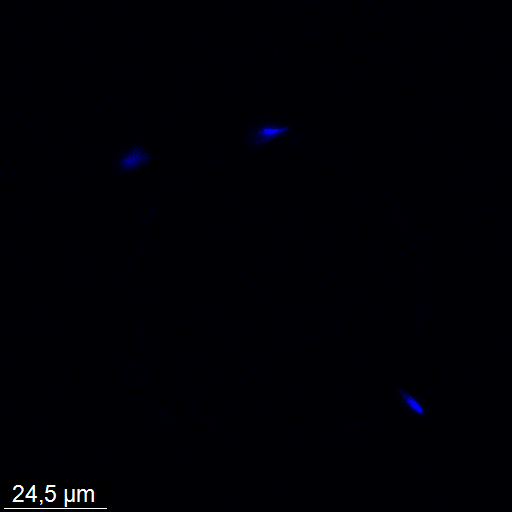

Supplement: Supplementary file 11 — Source data Fig. 6 [file 44319_2025_670_MOESM11_ESM.zip › Fig 6/Figure 6B/Fertilized slice 1/CD9GFP F3 stack_z24_ch00.tif]

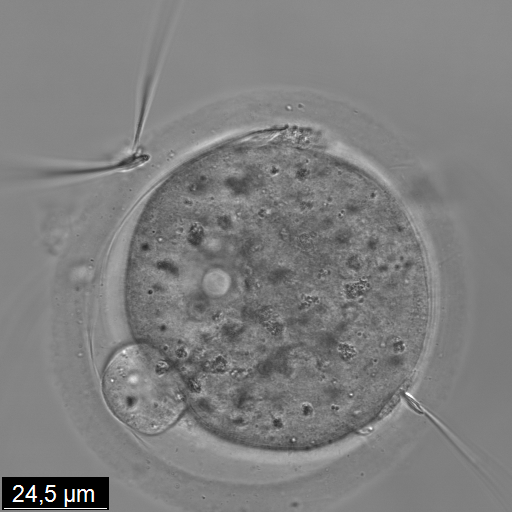

Supplement: Supplementary file 11 — Source data Fig. 6 [file 44319_2025_670_MOESM11_ESM.zip › Fig 6/Figure 6B/Fertilized slice 1/CD9GFP F3 stack_z24_ch01.tif]

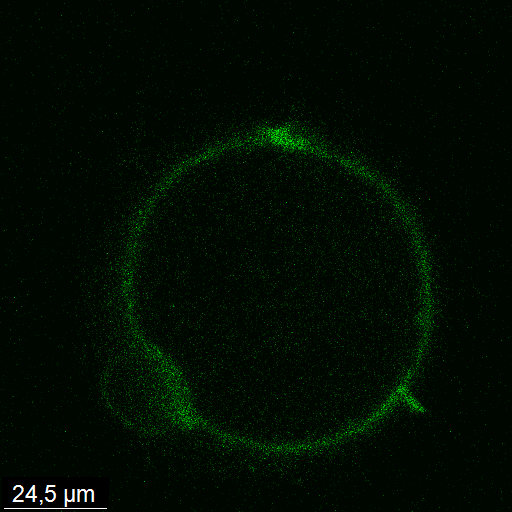

Supplement: Supplementary file 11 — Source data Fig. 6 [file 44319_2025_670_MOESM11_ESM.zip › Fig 6/Figure 6B/Fertilized slice 1/CD9GFP F3 stack_z24_ch02.tif]

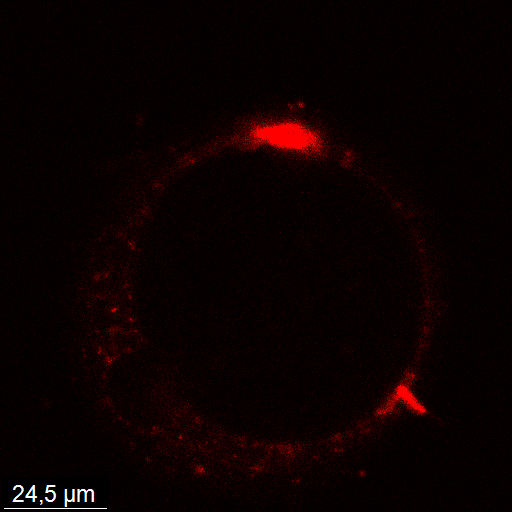

Supplement: Supplementary file 11 — Source data Fig. 6 [file 44319_2025_670_MOESM11_ESM.zip › Fig 6/Figure 6B/Fertilized slice 1/CD9GFP F3 stack_z24_ch04.tif]

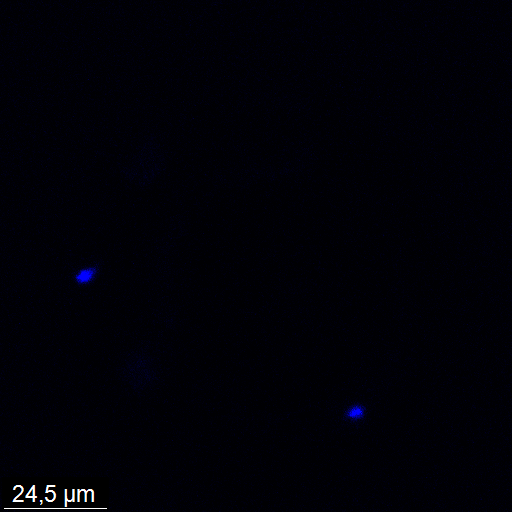

Supplement: Supplementary file 11 — Source data Fig. 6 [file 44319_2025_670_MOESM11_ESM.zip › Fig 6/Figure 6B/Fertilized Slice 2/CD9GFP F3 stack_z33_ch00.tif]

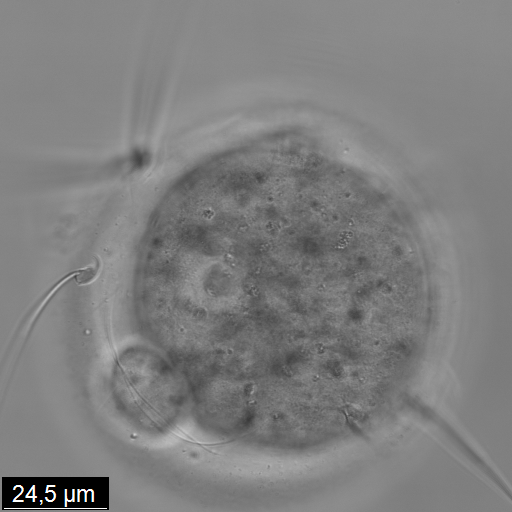

Supplement: Supplementary file 11 — Source data Fig. 6 [file 44319_2025_670_MOESM11_ESM.zip › Fig 6/Figure 6B/Fertilized Slice 2/CD9GFP F3 stack_z33_ch01.tif]

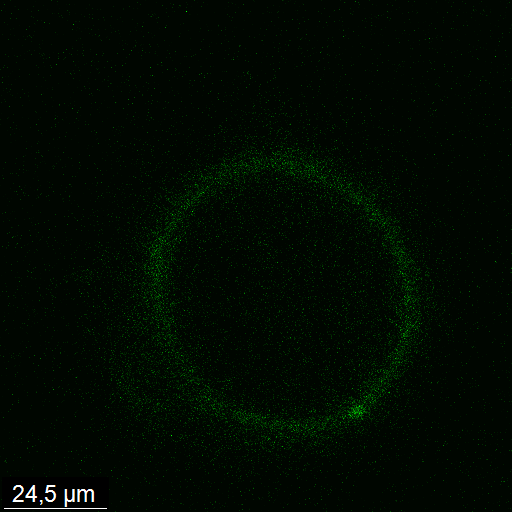

Supplement: Supplementary file 11 — Source data Fig. 6 [file 44319_2025_670_MOESM11_ESM.zip › Fig 6/Figure 6B/Fertilized Slice 2/CD9GFP F3 stack_z33_ch02.tif]

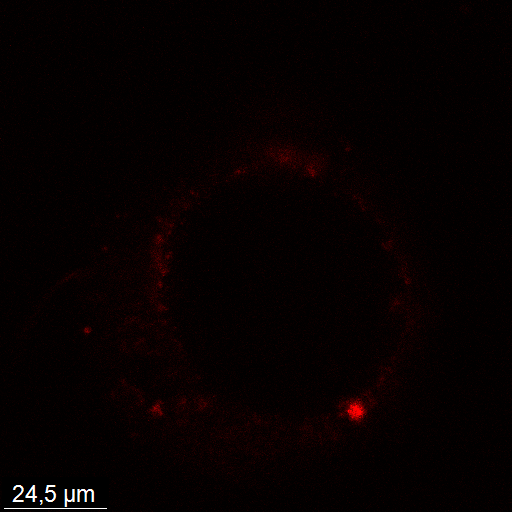

Supplement: Supplementary file 11 — Source data Fig. 6 [file 44319_2025_670_MOESM11_ESM.zip › Fig 6/Figure 6B/Fertilized Slice 2/CD9GFP F3 stack_z33_ch04.tif]

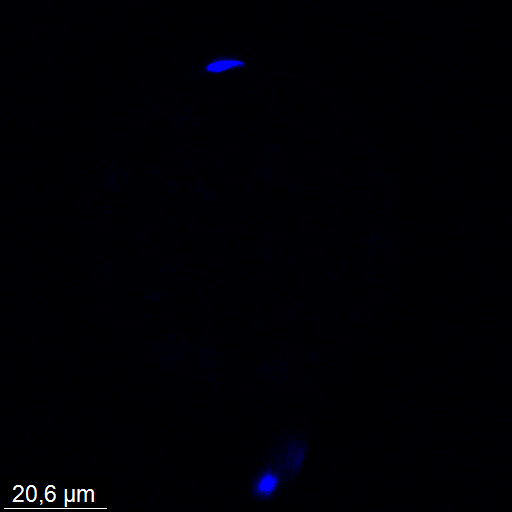

Supplement: Supplementary file 11 — Source data Fig. 6 [file 44319_2025_670_MOESM11_ESM.zip › Fig 6/Figure 6B/Unfertilized Slice 1/CD9GFP NF1 stack_z11_ch00.tif]

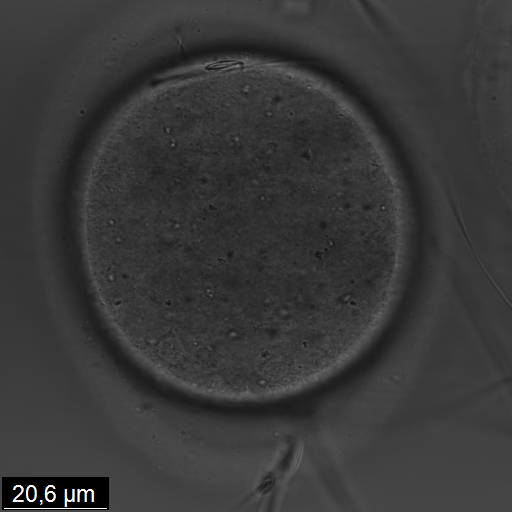

Supplement: Supplementary file 11 — Source data Fig. 6 [file 44319_2025_670_MOESM11_ESM.zip › Fig 6/Figure 6B/Unfertilized Slice 1/CD9GFP NF1 stack_z11_ch01.tif]

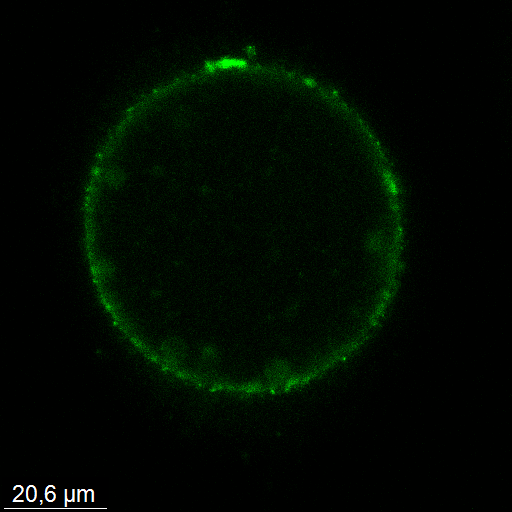

Supplement: Supplementary file 11 — Source data Fig. 6 [file 44319_2025_670_MOESM11_ESM.zip › Fig 6/Figure 6B/Unfertilized Slice 1/CD9GFP NF1 stack_z11_ch02.tif]

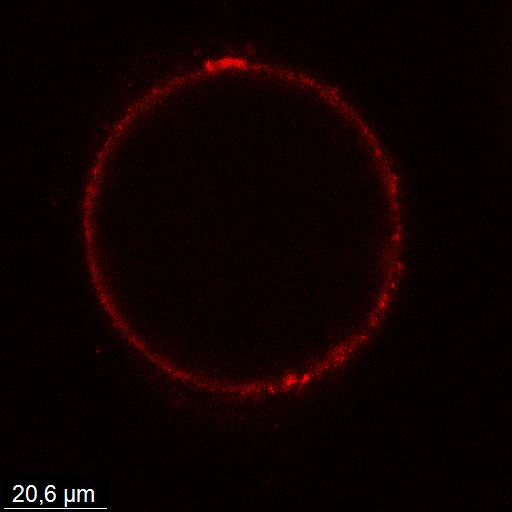

Supplement: Supplementary file 11 — Source data Fig. 6 [file 44319_2025_670_MOESM11_ESM.zip › Fig 6/Figure 6B/Unfertilized Slice 1/CD9GFP NF1 stack_z11_ch04.tif]

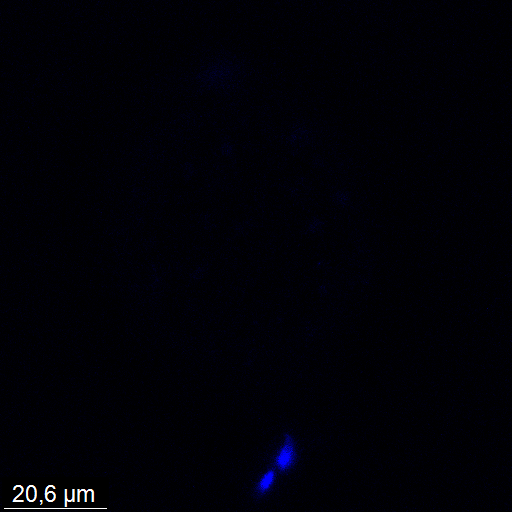

Supplement: Supplementary file 11 — Source data Fig. 6 [file 44319_2025_670_MOESM11_ESM.zip › Fig 6/Figure 6B/Unfertilized Slice 2/CD9GFP NF1 stack_z06_ch00.tif]

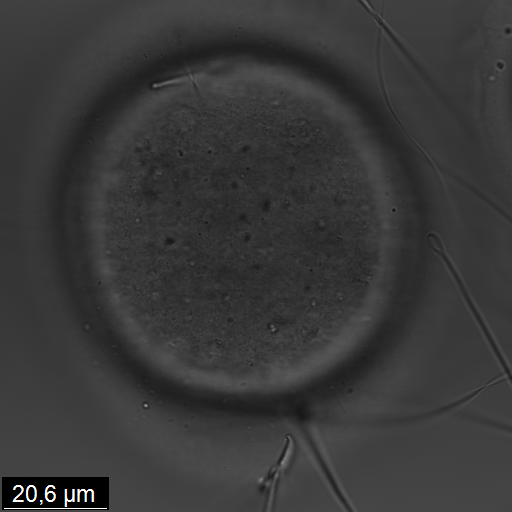

Supplement: Supplementary file 11 — Source data Fig. 6 [file 44319_2025_670_MOESM11_ESM.zip › Fig 6/Figure 6B/Unfertilized Slice 2/CD9GFP NF1 stack_z06_ch01.tif]

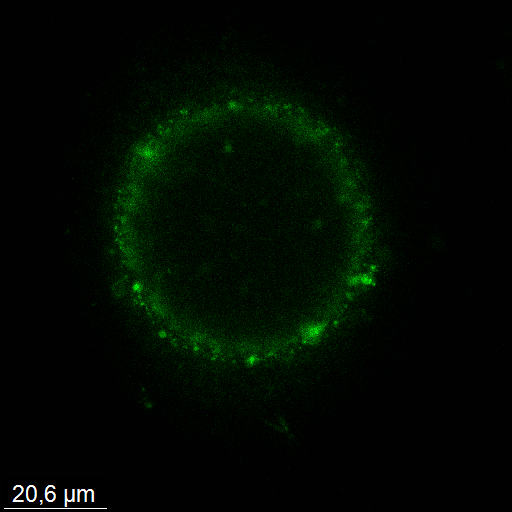

Supplement: Supplementary file 11 — Source data Fig. 6 [file 44319_2025_670_MOESM11_ESM.zip › Fig 6/Figure 6B/Unfertilized Slice 2/CD9GFP NF1 stack_z06_ch02.tif]

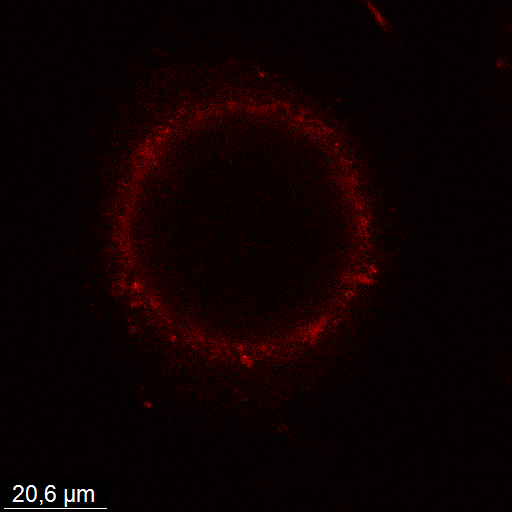

Supplement: Supplementary file 11 — Source data Fig. 6 [file 44319_2025_670_MOESM11_ESM.zip › Fig 6/Figure 6B/Unfertilized Slice 2/CD9GFP NF1 stack_z06_ch04.tif]
